# Sahaj Samadhi Meditation versus a Health Enhancement Program for depression in chronic pain: protocol for a randomized controlled trial and implementation evaluation

**DOI:** 10.1186/s13063-020-04243-z

**Published:** 2020-04-07

**Authors:** Abhimanyu Sud, Michelle L. A. Nelson, Darren K. Cheng, Alana Armas, Kirk Foat, Michelle Greiver, Fardous Hosseiny, Joel Katz, Rahim Moineddin, Benoit H. Mulsant, Ronnie I. Newman, Leon Rivlin, Akshya Vasudev, Ross Upshur

**Affiliations:** 1 Department of Family and Community Medicine, Faculty of Medicine, University of Toronto, Toronto, ON Canada; 2 Institute of Health Policy, Management and Evaluation, Dalla Lana School of Public Health, University of Toronto, Toronto, ON Canada; 3 Bridgepoint Collaboratory for Research and Innovation, Lunenfeld–Tanenbaum Research Institute, Sinai Health, Toronto, ON Canada; 4 London, ON Canada; 5 Department of Family and Community Medicine, North York General Hospital, Toronto, ON Canada; 6 Canadian Mental Health Association, Toronto, ON Canada; 7 Department of Psychology, Faculty of Health, York University, Toronto, ON Canada; 8 Department of Psychiatry, Faculty of Medicine, University of Toronto, Toronto, ON Canada; 9 Research and Health Promotion Department, Art of Living Foundation (North America), Saint-Mathieu-du-Parc, QC Canada; 10 Lifelong learning Institute, Health Professions Division, Nova Southeastern University, Davie, FL USA; 11 Rivlin Medical Group, Mississauga, ON Canada; 12 Emergency Medicine, Humber River Hospital, Toronto, ON Canada; 13 Department of Psychiatry, Western University, London, ON Canada; 14 Department of Neuroscience, Western University, London, ON Canada; 15 Geriatric Mood Disorders Lab, Lawson Health Research Institute, Parkwood Institute of Mental Health Care, London, ON Canada; 16 Dalla Lana School of Public Health, University of Toronto, Toronto, ON Canada

**Keywords:** Chronic pain, Depression, Meditation, Education, Randomized controlled trial (RCT), Implementation, Hybrid study, Sahaj Samadhi, Opioids

## Abstract

**Background:**

Despite the high prevalence of comorbid chronic pain and depression, this comorbidity remains understudied. Meditation has demonstrated efficacy for both chronic pain and depression independently, yet there have been few studies examining its effectiveness when both conditions are present concurrently. Furthermore, while meditation is generally accepted as a safe and effective health intervention, little is known about how to implement meditation programs within or alongside the health care system.

**Methods:**

We will conduct a hybrid type 1 effectiveness–implementation evaluation. To measure effectiveness, we will conduct a randomized controlled trial comparing Sahaj Samadhi Meditation and the Health Enhancement Program in 160 people living with chronic pain, clinically significant depressive symptoms, and on long-term opioid therapy. Changes in depressive symptoms will be our primary outcome; pain severity, pain-related function, opioid use, and quality of life will be the secondary outcomes. The primary end point will be at 12 weeks with a secondary end point at 24 weeks to measure the sustainability of acute effects. Patients will be recruited from a community-based chronic pain clinic in a large urban center in Mississauga, Canada. The meditation program will be delivered in the clinical environment where patients normally receive their chronic pain care by certified meditation teachers who are not regulated health care providers. We will use a mixed-methods design using the multi-level framework to understand the implementation of this particular co-location model.

**Discussion:**

Results of this hybrid evaluation will add important knowledge about the effectiveness of meditation for managing depressive symptoms in people with chronic pain. The implementation evaluation will inform both effectiveness outcomes and future program development, scalability, and sustainability.

**Trial registration:**

ClinicalTrials.gov: NCT04039568. Registered on 31 July 2019.

## Contributions to the literature

This is the first trial examining meditation to improve depression and opioid use in chronic pain.Evidence supporting nonmedication interventions is needed, including how to better integrate them into health care systems. Existing community-based certified teachers can make meditation more accessible and reduce the strain on formal health care services.

## Background

Chronic pain is a common and disabling health condition with significant population-level impacts [1]. The prevalence of chronic pain internationally is approximately 15–20%. In the USA, total health costs and indirect costs such as lost productivity are estimated to be $635 billion per year [2–4]. Comorbid mental illnesses are common in patients with chronic pain, with rates as high as 60% [5] compared to 10–15% in age-matched controls. This comorbidity is associated with poorer overall health outcomes, greater health care utilization, and higher rates of suicide [5]. Living with chronic pain and having a mental illness is also associated with an increased risk of being prescribed an opioid [6], being maintained on long-term opioid therapy (LTOT) [7], and being on higher doses of opioids [8]. Additionally, those with a mental illness who are prescribed opioids have a higher risk of developing a substance use disorder and of overdosing [9], with psychotropic medications prescribed for chronic pain comorbidities compounding the risk of overdose [10–12]. Adding to the complexity of these issues, there is evidence that LTOT is by itself a risk factor for developing de novo depression [13].

Despite the scale and impacts of this comorbidity, there is little evidence-based guidance for the management of mental illnesses in patients with chronic pain. A recent clinical practice guideline for the management of chronic pain [14] has a single recommendation for the management of depression in chronic pain, citing only one controlled trial that investigated a combination of antidepressant therapy and an education program. Clinical experience suggests that pharmacologic approaches to symptom and disorder management can be challenging to implement for people on LTOT due to polypharmacy, a particular challenge for older people [15]. In addition, pharmacotherapy with LTOT carries a risk of overdose and accidental death. Given the complexity and risk associated with pharmacotherapy in patients with chronic pain, there is a need for increased evidence for, and availability of, nonpharmacological treatments to improve quality of life, as well as to decrease pain severity and harms from opioids [16].

A substantial body of evidence suggests that meditation is a promising nonpharmacological intervention for chronic pain. In terms of efficacy, a recent systematic review demonstrated moderate effects for meditation and pain (effect size, 0.33), depression (effect size, 0.30) and anxiety (effect size, 0.38) [17]. Several systematic reviews examining meditation interventions specifically in the chronic pain population have reported small but consistent improvements in pain, function and depression [18–21]. Meditation is commonly sought out by diverse people living with depression and chronic pain and the intervention is well received in these populations [22–24]. *Sahaj Samadhi* Meditation (SSM), a type of automatic self-transcending meditation, is easy to learn and master [25] and its processes can lead to stress reduction and deep relaxation [26]. A randomized controlled trial (RCT) comparing this type of meditation to an active control in adults >55 years of age showed clinically significant reductions (48%) in depressive symptoms [27]. SSM has yet to be evaluated for its effects on depression in people with chronic pain.

Despite evidence of the effectiveness of meditation programs for chronic pain and depression, this intervention has not been widely implemented in North American health systems. The reasons for this are not well understood but may include negative perceptions about the effectiveness of meditation, lack of reimbursement for its delivery and perceived costs, or a mismatch between the holistic and lifestyle approach of meditation and the biomedical orientation that typify contemporary health services. Delivering complementary interventions where patients are usually seen for clinical care by a health professional, such as a physician, nurse, psychologist or physiotherapist, is one approach that has been used to improve the uptake of these interventions. This approach may improve access to these interventions by improving trustworthiness, physical access and familiarity. It may also address human resourcing by providing a means for reimbursement via traditional provider payment models. However, the medicalization of these interventions may have unintended negative consequences at both the intervention and health service level. For instance, it could be argued that health professionals need to focus on their scope of practice and that they do not have time for additional training or to deliver meditation programs. We also speculate that meditation delivered in a clinical environment by a biomedically trained health care provider may change its nature and its effects. There are scores of expert community-based meditation teachers who could provide this intervention without having to divert scarce human health resources.

This approach is in keeping with a stepped-care model of mental health service delivery based on the principle of ‘least burden’, whereby service providers triage patients into the least intensive service that is likely to meet their needs and be effective [28]. The lower tiers include community-based supports that rely on nonspecialists or peers rather than on formal health care resources. However, these lower-tier services are currently underfunded and underutilized in Canada. Investing in them could enhance mental health promotion and reduce the strain on access to higher-tier acute care services [29].

In this context, we posit that there is a need for evidence supporting the use of meditation as a health intervention for those living with chronic pain and comorbid depression. This includes evidence on how to implement such programs in the context of contemporary health systems. These dual and somewhat competing goals can be accomplished with a hybrid design that combines evaluations of both health outcomes of an intervention and the process of its implementation. The scale of the chronic pain and depression comorbidity and its contemporary association with the opioid crisis suggest the need for a more rapid development of usable knowledge which hybrid designs are well positioned to do [30].

This protocol article describes the MEDOTATE study (Meditation for depression and opioid use in chronic pain: an RCT and implementation evaluation), a hybrid type 1 study focusing on the effectiveness of SSM for patient outcomes related to depression, pain, function and quality of life, and which also evaluates the implementation of a collaboration between a community-based chronic pain clinic and certified meditation teachers to provide this intervention. This article describes the study procedures and discusses key methodological decisions and challenges for a hybrid design for a meditation intervention.

**Fig. 1:**
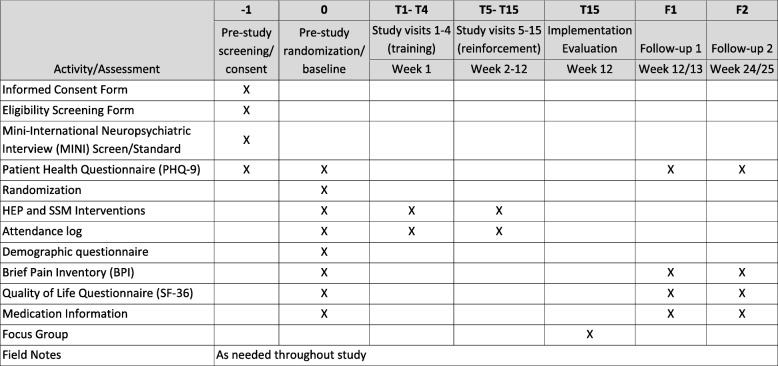
Protocol schedule of assessments and interventions. *Abbreviations:* *BPI* Brief Pain Inventory, *HEP* Health Enhancement Program, *MINI* Mini-International Neuropsychiatric Interview, *SF-36* 36-item Short Form survey, *SSM* Sahaj Samadhi Meditation

We report the protocol for the effectiveness trial using the Standard Protocol Items: Recommendations for Interventional Trials (SPIRIT) statement [31], and describe the intervention using the Template for Intervention Description and Replication (TIDieR) checklist and guide [32]. We report the protocol for the implementation evaluation using the Standards for Reporting Implementation Studies (StaRI) statement [33]. See Fig. 1 for the completed SPIRIT figure presenting the protocol schedule of assessments and interventions. The completed World Health Organization Trial Registration Data Set can be found in Additional file 1.

## Methods

This study was developed to examine the clinical effectiveness and implementation of an intervention using SSM for older adults living with chronic pain and comorbid depression. The project is being conducted at a community-based chronic pain clinic in a major urban center in Ontario, Canada, with the collaboration of a community-based, non-profit meditation provider. Design of the program included input from a national mental health service delivery and advocacy organization and a person with lived experience, and both will also be integrated in oversight of program delivery and evaluation. The study was approved by the research ethics board of both Mount Sinai Hospital (19-0163-A) and the University of Toronto (00038321); it is funded by the Canadian Institutes for Health Research Opioid Crisis Evaluation Grant (EO1-162072). The trial is registered at clinicaltrials.gov (NCT04039568; https://clinicaltrials.gov/ct2/show/NCT04039568).

SSM is a standardized and manualized meditation program delivered in community settings by certified non-clinician meditation teachers (https://www.artofliving.org/sahaj-samadhi-meditation). Program delivery and teacher certification is conducted by Art of Living (AOL), an international non-profit charitable organization which reaches across 150 countries worldwide. Teachers are required to complete at least 1200 h of personal SSM practice and an intensive teacher training program. SSM is taught in the community through a course called The Art of Meditation, which is a 6-h program over 3 consecutive days without any prerequisites.

Besides its established clinical efficacy for a variety of conditions, meditation carries other advantages as a potential population health intervention. Experience from AOL suggests that the program can be delivered effectively by expert nonclinicians or lay providers (often volunteers); there are many meditation teachers who can be enlisted to provide this intervention, and fidelity of the intervention can be maintained over different sizes of groups. These aspects of SSM suggest the potential for scalability to meet not just clinical needs but also population health needs.

Despite this potential for SSM to beneficially impact the lives of people with chronic pain, mental illness, and using prescribed opioids, there are a variety of real and perceived barriers specifically around reach and access. Some possible, and as yet uninvestigated, barriers include: lack of awareness of the existence of the program by the target audience; lack of awareness of the potential benefits or the nature of the program by the target audience; the monetary cost of the program; physical access to community sites where the program is typically offered; and skepticism of the health benefits by trusted health care providers.

Considering the overall context and the barriers to access, AOL, the Canadian Mental Health Association (CMHA), the Rivlin Medical Group (a community-based chronic pain management clinic), and an academic health services research group are collaborating to develop, deliver and evaluate MEDOTATE. MEDOTATE offers a modified version of SSM by the same teachers as in the community but co-locates its delivery within the context of formal clinical chronic pain care.

As a means of overcoming access barriers, MEDOTATE will offer SSM in the same clinical space and recruitment will occur through the administrative processes that patients are accustomed to using for their routine medical care. SSM will be delivered by three experienced local meditation teachers from AOL, selected by the AOL research director. The AOL research director will also provide ongoing training and troubleshooting for the meditation teachers. Clinic staff and clinicians will also be educated about the program to facilitate service delivery in multiple 1-h in-person informational sessions, email memoranda, and one-on-one discussions.

### Study objectives

There are two broad evaluation objectives. The primary objective is to evaluate the effectiveness of SSM. This evaluation will aim to answer the question: “is SSM effective in improving depressive symptoms and associated variables in chronic pain?”. For this effectiveness objective, we have selected change in depressive symptoms as the primary outcome because of the strength of the pilot data used to inform evaluation design. The secondary effectiveness outcomes will be reduction in pain severity and pain interference, reduction in opioid use, and improvement in quality of life.

The second objective is to evaluate the implementation of SSM in the specific setting of a community-based chronic pain clinic. This part of the evaluation will aim to answer the following questions: “how was SSM implemented in this setting?”, “what were the barriers and facilitators to implementation?” and “how can SSM be translated to other settings?”. This implementation evaluation will help to interpret the effectiveness outcomes and it will be used to inform both implementation and effectiveness outcomes in other, real-world settings such as in multi-provider primary care sites or multi-provider community care sites.

### Study design

MEDOTATE has a hybrid type 1 evaluation design. Hybrid designs aim to blend effectiveness and implementation research to “improve the speed of knowledge creation and increase the usefulness and policy relevance of clinical research”, both of which are imperatives for this proposed evaluation [30]. Specifically, hybrid type 1 designs aim to primarily test the effectiveness of an intervention while gathering information relevant to real-world implementation. In type 1 designs, implementation evaluation is secondary to the primary goal of effectiveness evaluation. This is appropriate given the nature and priorities of the evaluation objectives outlined above where effectiveness of the intervention in this population is not yet established.

Curran et al. [30] note the inherent tensions between effectiveness and implementation evaluations, especially in that they differ in what they test (a clinical intervention versus an implementation strategy), their typical units of randomization and analysis (patient versus organization), and the kinds of outcomes they seek. An important limitation of our approach is that it is not a true evaluation of implementation as there is no a priori commitment on the part of the community-based chronic pain clinic to continue delivering this program beyond the trial period. Despite similar limitations, other groups have successfully executed the implementation component of type 1 hybrid evaluations [34].

Given the character of the hybrid design, a mixed-methods approach to evaluation is appropriate. We will use a QUAN(qual) embedded design, as described by Creswell and Plano Clark [35]. The focus of the evaluation will be on effectiveness in the form of a single-site, single-blinded (investigator and clinician), 12-week RCT powered for the depression outcome and measuring changes in pain, medication use and quality of life as secondary outcomes. Participants will undergo 1:1 randomization to the SSM intervention or to an active control called the Health Enhancement Program (HEP). The two interventions will be run in groups of 10–15 participants on a rolling monthly basis. This will decrease intervention wait times and allow for timely access to the offered interventions and their potential benefits. Additionally, ethics approval for this study was granted under the title “Alternative treatments for depression in chronic pain” to avoid biasing participants with investigators’ hypotheses and to improve retention in the control arm.

Embedded within, and secondary to, this RCT will be an implementation evaluation using both quantitative and qualitative approaches. The implementation evaluation will focus on assessing reach, dose, satisfaction and understanding program implementation facilitators and barriers.

### Clinical effectiveness

#### Study setting

The target population will be midlife and older patients with chronic pain attending a community-based pain clinic with comorbid depression and on LTOT. This will include patients both newly referred to the clinic and existing patients.

#### Eligibility criteria

Specific inclusion and exclusion criteria and their associated rationales are detailed in Table 1.

**Table 1:** Eligibility criteria

Criteria	Rationale
***Inclusion criteria***	
1. >45 years of age; chronic pain (pain ≥3 months duration in any body region by self-report)	Older people with chronic pain are disproportionately affected by harms from prescribed opioids such as overdose and death [36]
2. On LTOT (any opioid at any dose for ≥3 months by self-report)	Known contribution of LTOT to depression and secondary outcome of reducing opioid use/dose [13]
3. Comorbid depressive symptoms of mild to moderate severity (PHQ-9 score 10–19)	People with subsyndromal depressive symptoms (PHQ-9 score 5–9) are less likely to show an effect from any intervention
4. Understanding of English language; able to sit for 20–25 min without significant discomfort; be willing and able to attend all four training sessions of SSM/HEP, as well as 75% of follow-up sessions	Ensure ability to participate in interventions which will be delivered in English only and mostly in a seated position
***Exclusion criteria***	
1. Psychiatric conditions other than depression, including substance use disorder, psychosis and cognitive impairment as established by the MINI; severe depression (PHQ-9 ≥20) and risk of imminent suicide as per MINI and PHQ-9; noncorrectable, clinically significant sensory impairment; acutely unstable physical illnesses, including delirium or acute cerebrovascular or cardiovascular events within the last 6 months; a terminal medical diagnosis with prognosis of less than 12 months	People with these attributes are theoretically less likely to benefit from the group meditation intervention and/or HEP control condition. People with severe depression (i.e., PHQ-9 ≥20) may need a standard intervention (e.g., psychotherapy, antidepressant medications). In certain cases, a patient’s symptoms may worsen (e.g., patients with psychosis) or the patient may be too frail to complete the study (e.g., medically unstable patients) [37]
2. Currently practicing any form of mind–body intervention	To limit confounding variables
3. Inability to provide informed consent	As per ethical norms for research involving human subjects

*Abbreviations: HEP* Health Enhancement Program, *LTOT* Long-term opioid therapy, *MINI* Mini-International Neuropsychiatric Interview, *PHQ-9* Patient Health Questionnaire, *SSM* Sahaj Samadhi Meditation

#### SSM intervention

The MEDOTATE program includes SSM training by a certified meditation teacher for 4 consecutive days (2 h/day) in the first week, followed by 75-minute (min)/week reinforcement sessions for 11 weeks. Following the protocol of a previous RCT [36], this is a longer version of the program that is taught in the community to accommodate the greater illness burden in the study population as compared to the community. On day 1, participants learn the nature of meditation, and then undergo personal guided meditation. Training on days 2 to 4 includes understanding the nature of the mind and the thoughts arising from it, guided meditation by the teacher, and discussions of what is appropriate and inappropriate meditation practice. Participants learn how to respond to experiences that arise in meditation, discuss what enhances or detracts from effective meditation, and review methods for meditating at home. Throughout the 12 weeks, participants will also be encouraged to practice twice daily at home for 20 mins per session. Weekly 75-min reinforcement sessions will include 20 mins of guided meditation practice, and then focus on participants’ experiences with meditation during the week, additional observations, and a review of relevant knowledge to support their practice at home.

#### Control intervention

HEP has been designed and used as a manualized active control in other meditation-based intervention trials [37–39]. HEP controls for several nonspecific factors found in a meditation group, including group support and morale, behavioral activation, reduction of stigma, facilitator attention, treatment duration, and time spent on at-home practice (adapted from a published manual [40]). HEP will be tailored to be structurally equivalent to SSM with similar sized groups, meeting schedule, total contact hours, amount of home practice and encouragement to keep practice logs. Participants will learn about health promotion, healthy diet, music and exercise, but will not learn meditation. In HEP, participants get the support of a group and facilitator, and talk through and attempt to implement positive health-enhancing life changes. HEP will be delivered by regulated health care providers not associated with the pain clinic and with formal training by an experienced HEP trainer. There will be no active intervention with respect to prescribed medications during the study for either arm of the study.

#### Outcome measures

For the RCT, change in depressive symptoms (using the nine-item Patient Health Questionnaire (PHQ-9) scores) from baseline to 12 weeks has been chosen as the primary outcome of interest rather than change in opioid dosage for a number of reasons. Change in opioid dosage, while certainly a safety and public health-oriented outcome, is not a patient-centered outcome. Previous studies examining opioid tapering strategies have suffered from poor recruitment as well as selection bias, perhaps reflecting lack of motivation and interest amongst patients in reducing opioid doses [41]. This has been seen specifically in other studies of nonpharmacological interventions for opioid dose reduction [42]. Likewise, studies that have assessed changes in opioid dosage reduction in response to a meditation intervention have shown very small effect sizes [43]. A study powered to assess opioid dosage reduction as the primary outcome would have to be very large, which is unfeasible given the available resources for this study. The high burden of depressive symptoms in chronic pain, the important relationship between comorbid depressive symptoms and opioid use, and the established moderate effect sizes of SSM on depression from existing data suggest that change in depressive symptoms is an appropriate primary outcome measure for this study. Finally, this choice of outcome measures is commensurate with the IMMPACT recommendations for core outcome measures for chronic pain clinical trials [44]. Further details about the outcome measures are included in Table 2. Given the typical relapsing and remitting nature of depression, and also the loss of effect over time from behavioral interventions, we will repeat all outcome measures again at 24 weeks to assess the sustainability of effects observed acutely or for the emergence of effects over a longer period of time.

**Table 2:** Effectiveness primary and secondary outcome measures

Outcome measure	Rationale
Primary: change in nine-item Patient Health Questionnaire (PHQ-9) scores from baseline to 12-week follow-up and 24-week follow-up (continuous)	The PHQ-9 is a well-validated and widely used self-report scale used in depression and chronic pain clinical care and research. Reduction in a continuous outcome score is a more sensitive test than percent reduction in scores that have been reported in some depression studies [5, 45, 46]. A self-report scale is more feasible than an assessor-rated scale (e.g., Hamilton Depression Rating Scale (HAM-D17)) given the site characteristics
Secondary A: change in Brief Pain Inventory (BPI) from baseline to 12-week follow-up and 24-week follow-up (continuous)	The BPI is a validated self-report scale used in pain trials and clinical pain practice and is a core outcome measure per the IMMPACT recommendations [47]. Two independent measures are included—pain severity and pain interference with function—and both are clinically relevant outcomes
Secondary B: change in opioid dosage (reported in total daily morphine equivalents) from baseline to 12-week follow-up and 24-week follow-up (self-reported; continuous measurement with conversion to total daily morphine equivalents using standardized conversion tables)	Self-report of opioid dose via patient log is the most common measure of opioid use in clinical research trials [48]. Given that patients are on a variety of distinct opioids and that some patients may be on >1 formulation, there is a need for common reporting as provided by morphine equivalent doses
Secondary C: change in health-related quality of life (QoL; by the 36-item Short Form survey (SF-36)) from baseline to 12-week follow-up and 24-week follow-up (continuous measurement)	The SF-36 has been validated as a quality QoL measure in the chronic pain population and is recommended by IMMPACT [49]. It is included here as a secondary outcome with expected positive findings from pilot data collected in other meditation interventions, which demonstrate moderate-to-large effect sizes [50]. This measure is well-aligned to the intervention which is designed as a means of improving overall QoL more than as a disease treatment

In addition to these primary and secondary outcome measures, participants will also complete a baseline demographics survey that will collect information on age, gender, ethnic background, employment status, housing type, highest level of education achieved, smoking, caffeine intake, drug and alcohol use, duration of pain and pain diagnosis. Participants will also be asked about their psychiatric history including age of first contact with services for mental illness (and which illness), history of hospital admissions and the number of episodes of depression. Reasons for withdrawal, loss to follow-up, or removal from the study will be recorded.

#### Study timeline

**Fig. 2:**
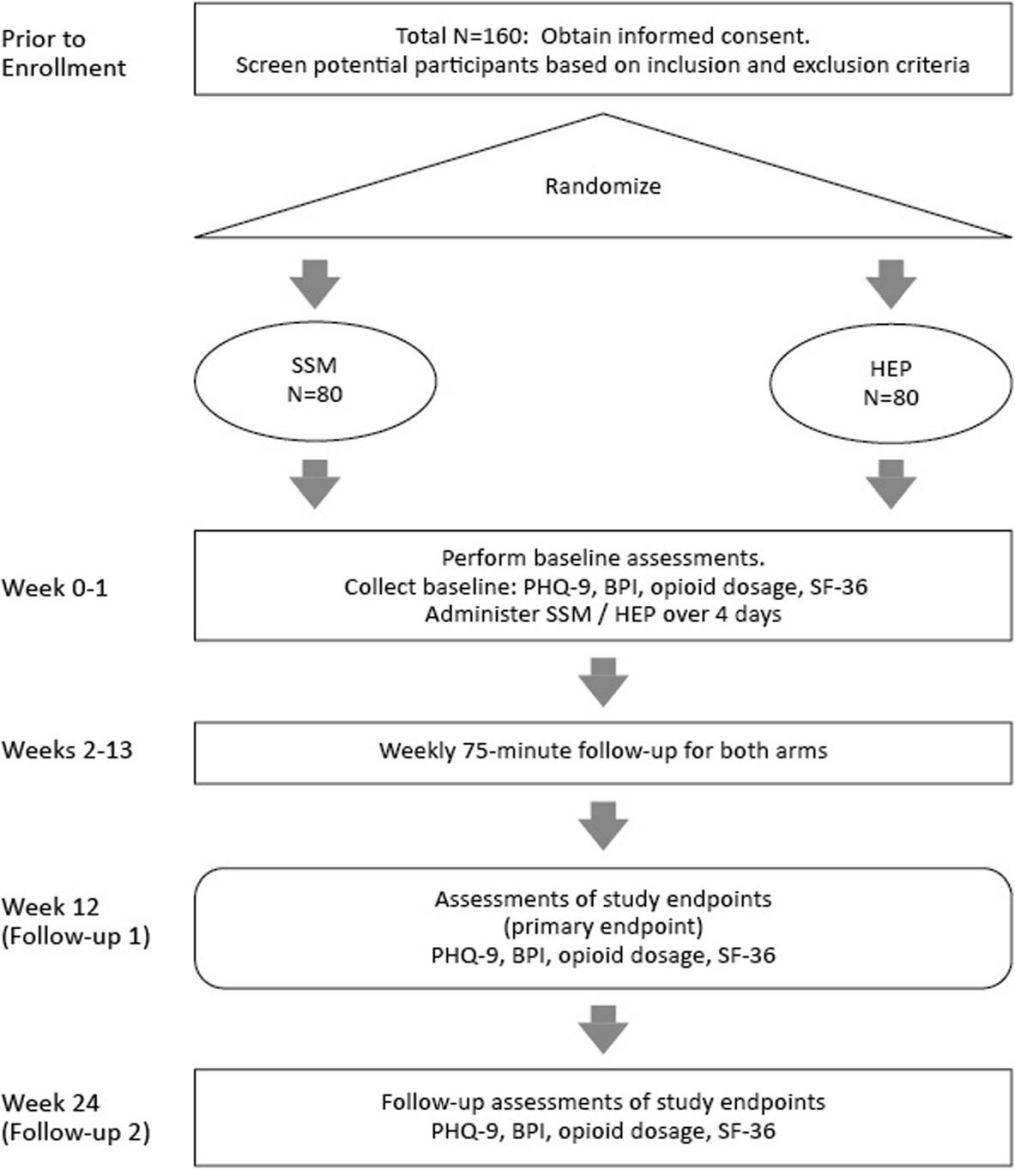
Effectiveness study flow diagram. *Abbreviations:* *BPI* Brief Pain Inventory, *HEP* Health Enhancement Program, *PHQ-9* Patient Health Questionnaire, *SF-36* 36-item Short Form survey, *SSM* Sahaj Samadhi Meditation

A flow diagram of study procedures is presented in Fig. 2.

#### Sample size calculation

A practice audit at the pain clinic in which the study will be conducted identified a high degree of comorbid depression and chronic pain in a random selection of 50 patients. Mean PHQ-9 score was 11 (standard deviation 5.7), where scores ≥10 indicate clinical depression. Sixty percent of patients had PHQ-9 scores ≥10 but only 4% had scores ≥20 (severe depression). This audit also identified three pools of patients from which subject participants can be recruited. These include: 1) new consults (30/week of which 38% meet inclusion criteria; *n* = 720 over an estimated 6-month recruitment period); 2) current patients on LTOT prescribed by physicians outside the clinic (*n* = 212); and 3) current patients on LTOT prescribed by clinic physicians (*n* = 194). The estimated total pool of eligible patients to recruit from over a 6-month period is thus about 1100.

In terms of estimated sample size, the primary outcome is continuous as measured by PHQ-9. The standard deviation of the PHQ-9 is 5 units and we anticipate a 2.5-unit difference at the end of the study between the intervention and control groups based on extrapolation of the pilot data [51]. This study is a two-arm RCT; therefore, we used a two-sample *t* test to calculate the sample size to have at least 80% power to detect differences in PHQ-9 scores of 2.5 or larger at 5% type 1 error. The resulting estimated sample size is 64 participants in each arm. We inflated the sample size by 25% to account for dropouts, which is reasonable given an attrition rate of 13% seen in the pilot study [36]. Therefore, we will recruit and randomize a total of 160 participants, with 80 participants in each arm.

#### Identification and recruitment

Patients at the clinic will be introduced to the study by clinic staff and they will be asked for their permission to be contacted by the research coordinator who will then obtain informed consent and screen those that are interested in participation. (See Additional file 2 for the RCT Participant Informed Consent Form.)

#### Allocation and blinding

The data management and randomization services available through the Lunenfeld–Tanenbaum Research Institute (LTRI), Mount Sinai Hospital–Sinai Health System, Toronto, will be used as a central repository to computer generate randomization codes and ensure concealment of randomization. A research coordinator not involved in recruitment or evaluations will contact patients with allocation information. This will ensure study staff and investigators are blinded to allocation. Study data will be collected and managed using REDCap [47] hosted at LTRI. REDCap is a web-based software platform designed to support data capture for research studies that is widely used by health researchers to significantly reduce data entry and study management errors to improve data fidelity. All study participants will be additionally blinded to the study hypotheses to prevent expectation bias. A second research coordinator will be trained by investigators for appropriate oversight of and instructions for completion of self-report scales by participants. Since it will not be possible for participants to be blind to their intervention status, they will be given detailed instructions not to share allocation information with blinded study staff and their clinicians.

#### Risks and safety

While rare [36], the most commonly reported adverse effects from SSM include mild anxiety caused by the relaxation itself. Other adverse effects that may be experienced include boredom and feeling mildly detached from one’s surroundings. Participants experiencing any other adverse effects will be advised to consult with their primary care physician, as well as to report them to the site medical director who will consult with medical co-investigators to determine if participants experiencing such adverse effects should be permitted to continue. If considered medically unsafe to continue, the blind will be broken for further clinical management of the adverse effect. There are also no known direct risks associated with the control arm of this study. However, participants will be assessed for suicidal ideation at each assessment visit. Any indication of a change in their overall mental health or risk of suicide will be reported immediately for further clinical assessment and management. Attendance and homework completion logs will be completed by participants and will be used to monitor adherence.

### Implementation evaluation

#### Outcome measures

For the implementation evaluation, we will use quantitative measures to assess reach and dose [45]. For reach, we will assess two factors. The first is whether the program is able to meet recruitment targets. As discussed above, based on a recent practice audit, we anticipate being able to recruit 20–30 patients per month (10-15 to each arm). This is an ambitious recruitment rate, but justifiable given the volume and types of patients seen at the study site. We will keep a log to measure the number of participants recruited per month and how these participants were recruited. The second aspect of reach will be to measure who chooses to participate in the program. The demographic and clinical characteristics of participants can help to inform recruitment strategies for future program implementations or trials. They have been informative in similar hybrid designs. As one example, Hagedorn et al. [34] found that their program regarding contingency management for substance use in an American veteran population had significantly over-reached into African–American populations and populations that had stimulant dependence. These kinds of findings can have important implications for implementation in practice settings.

The second quantitative measure of implementation will be of dose. Specifically, we will assess the amount of meditation time of the study participants. This will be captured using attendance logs for the 4-day program and once weekly reinforcement sessions. Since participants will also be encouraged to practice the learned meditation technique at home for a minimum of 20 mins per day, we will also ask participants to maintain a log of their home practice. They will be encouraged to complete these logs during the reinforcement sessions if they have not completed them at home.

**Fig. 3:**
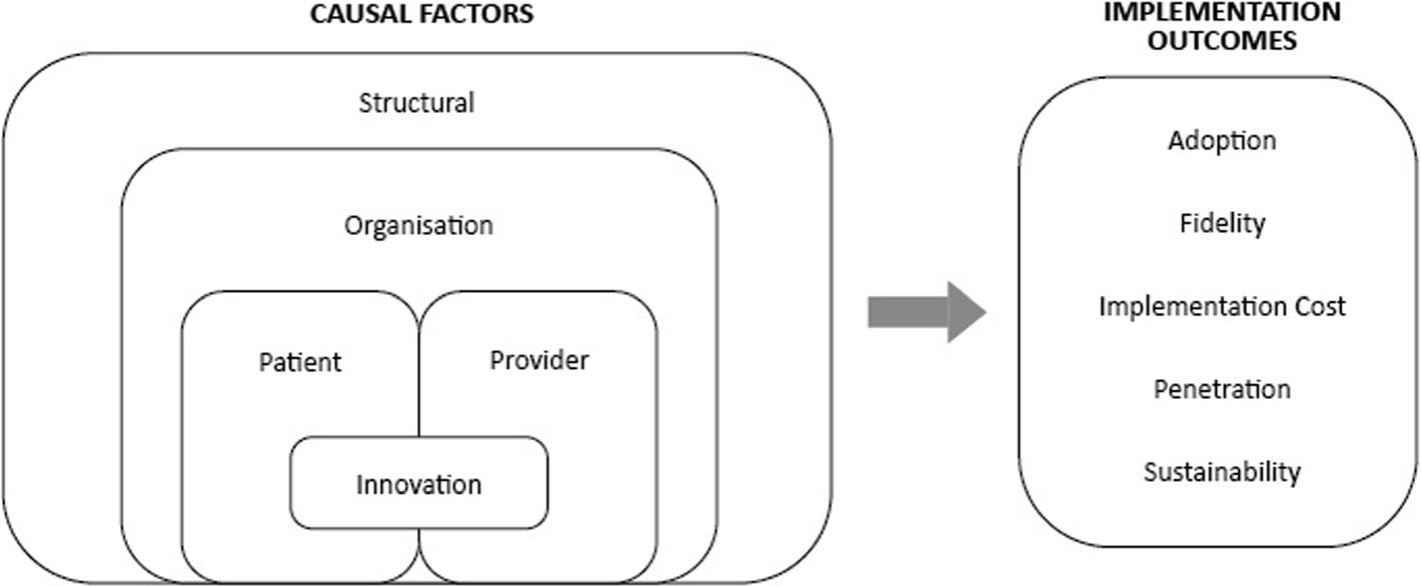
Multi-level framework predicting implementation outcomes [46]

The qualitative component of the implementation evaluation will assess primarily three factors: satisfaction with the program, facilitators and barriers of implementation, and transferability of the program to other settings, such as primary care clinics and nonclinical settings. Given that there will be robust data from the RCT regarding effectiveness, we will not focus on gathering qualitative data about effectiveness. We will use the multi-level framework of Chaudoir et al. [46] predicting implementation outcomes to guide the qualitative data collection methods and tools (Fig. 3).

#### Identification and recruitment

The multi-level framework is helpful in identifying the relevant perspectives that will be considered for the implementation evaluation. In this case, we can identify four key groups of participants who can provide multiple and unique perspectives on the various causal factors in the framework. First, patients in the SSM intervention arm will be invited to participate in a focus group on completion of their last in-person reinforcement session. They will have already learned and practiced meditation with other participants during approximately 20 h of group activities over 12 weeks. As such, there will be comfort and familiarity of sharing and reflecting in a group environment. This focus group will be able to provide particular insight into patient factors, but will also be queried for their important insights into the remaining factors. At the time of recruitment for the RCT, we will obtain consent for participation in the postintervention focus group. The focus group will be conducted immediately after an already scheduled reinforcement session to maximize participation.

Second, the three meditation teachers will be invited to participate in two focus groups, one prior to the launch of the program and one at completion. Again, this will be a group that will be familiar with each other, who will have worked together before and amongst whom there are unlikely to be important power imbalances that could interfere with focus group dynamics. Evidently, they will have unique perspectives on the provider factor, but also specifically on the innovation. Since they will have a wealth of experience delivering SSM in other settings, particularly community and sometimes other medical settings, we can draw on this experience to better understand the implementation of SSM in this specific context. Specifically, it will be important to query the tension between fidelity and adaptation across these various contexts [48]. While SSM is a standardized and manualized intervention, it is also delivered in a dynamic social setting so there may be important nuances in its delivery. The meditation teachers will also complete a short questionnaire at the end of each cohort cycle. The questionnaire will gather insights on the nuances in delivering SSM during the study period, and aid in determining the fidelity of the program. The meditation teachers will be contacted by telephone or email, and consent for participation will be obtained in person at the time of the focus groups.

Third, clinic administrative staff will be queried. They will have supported the implementation and execution of the program due to their integral involvement in patient recruitment and support of the delivery of the interventions. They will be able to provide key insights into organization factors that enable or hinder the implementation of the program.

We will also collect data from the clinical staff, which includes physicians from a variety of disciplines (family medicine, anesthesia, sports medicine and orthopedic surgery), nursing staff, and a chiropractor and a physiotherapist. Their perspectives will be particularly helpful in informing organization and structural factors.

At the completion of the RCT we will conduct separate focus groups for the administrative and clinical staff. Consent for participation will be obtained individually and in person. Since not all clinic staff are present every day, we will run two to three focus groups at convenient times at the clinic to maximize participation by this group.

In addition to the focus groups, we will conduct semistructured interviews with the administrative and clinical leads for the study. The interviews will take place prior to the launch and again at the completion of the study. We chose to conduct interviews with these individuals to allow for more in-depth discussions of their insights on the implementation of the program since these individuals will be most involved in supporting the delivery of the interventions. Also, including the leads in the focus groups may introduce bias as the other administrative and clinical staff may rely on the leads to answer, or tailor their answers to match the leads’ responses. As with the other staff, we will obtain consent for participation individually and in person. The interviews will be held at the clinic and at a convenient time for the leads.

Table 3 provides a sample of focus group and interview questions for all of the participants described above, and indicates some causal factors and implementation outcomes each question aims to address based on the framework from Chaudoir et al. [46]

**Table 3:** Sample of interview and focus group questions

Participant group	Question	Causal factor(s)	Implementation outcome(s)
SSM participants	*Postprogram* Why did you choose to participate? a. Would you have done this without the clinic suggesting it? Why or why not? What was your experience like learning meditation at the clinic? a. Tell us about something you enjoyed about learning meditation in this setting b. Is there anything you would change about this program if it were to continue in the clinic? Can you tell us why you would change this? c. Did you experience any challenges learning meditation at the clinic?	• Patient • Innovation • Organization	• Adoption • Sustainability
Meditation teachers	*Preprogram* Will you prepare anything differently than for a program in the community? *Postprogram* What was your experience of teaching in a medical setting? a. Did this setting impact how you taught the program? b. Were there any modifications to the program based on the setting? If so, which ones? c. What was your experience of setting up and running the program in the clinic? (logistics)	• Provider • Innovation • Organization	• Fidelity • Adoption • Sustainability
Administrative staff	*Postprogram* Did you face any challenges over the course of the program? a. What do you wish to have known prior to the program starting? b. What clinic resources were used to implement the program? Were they sufficient?	• Organization	• Implementation cost • Sustainability
Clinical staff	*Postprogram* What would you need to recommend the meditation program to your patients? a. Would you refer patients to the program if it continued in the clinic? b. What factors would you consider in referring?	• Organization • Structural	• Penetration • Sustainability
Administrative and clinical leads	*Preprogram* What are your expectations of this program? a. What challenges do you anticipate? b. What are your thoughts on this type of program running in a clinical setting? c. What are your thoughts on offering this type of program to the patient population in your clinic? *Postprogram* How were you involved in the program? c. Was this different from what you expected at the beginning of the program? d. If it changed, when and why did it change? e. How did your involvement in the program impact your regular role in the clinic?	• Organization • Structural	• Adoption • Implementation cost • Sustainability

*Abbreviations: SSM* Sahaj Samadhi Meditation

### Data collection

All data collected will be recorded using unique participant identifiers (IDs) to anonymize collected information. The effectiveness outcome measures are based on self-report and they will be collected at baseline and 12-week and 24-week assessment visits. They will be completed with assistance from a research coordinator blinded to participant allocation. Implementation evaluation outcomes (collected pre- and postprogram from clinic staff and SSM teachers, and postintervention from SSM participants) will be completed by a study staff. Qualitative implementation measures will be audio-recorded, transcribed verbatim, and anonymized. The final dataset will be accessible by all study investigators.

### Privacy, confidentiality and security

All questionnaires and forms will be completed digitally using REDCap and stored on a secure server based at LTRI. Following completion of a data transfer agreement, data in paper format (questionnaires) will be transported in a locked briefcase from the clinic to LTRI and will be stored in a locked cabinet for the duration of the study and discarded via secure shredding after a period of 7 years. All data will be coded with unique participant IDs to protect patient confidentiality, and documents linking participants to their IDs will be stored on a secure database at LTRI for the duration of the study. The code key with identifying data will only be accessible to research coordinators responsible for recruitment, assessment and scheduling, unless a participant’s health is at risk and unblinding is required due to extenuating circumstances. All the focus groups and interviews will be audio-recorded and transcribed verbatim. The transcripts will be read against the audio-recordings to remove identifiable information and to check for accuracy. All audio files and electronic copies of the transcripts will be transported on encrypted USB drives or audio-recording devices in a locked briefcase to LTRI and stored on the secure server for the duration of the study. Any hard copies of the transcripts will be stored at LTRI in a locked cabinet. After a period of 7 years all digital files will be permanently deleted, and hard copies will be discarded via secure shredding. The consent form informs participants that the research team and research ethics boards will have access to study data where relevant, and that they may withdraw from the study and request to have their study information removed at any time. No biological specimens will be collected.

### Data analysis

#### Effectiveness

The primary and secondary outcomes (PHQ-9 score, Brief Pain Inventory, opioid equivalent dose, and 36-item Short From survey) will be measured at baseline and at 12 and 24 weeks. We will examine the distribution of primary and secondary outcomes for normality. If required, appropriate transformation (for example Box–Cox transformation) will be applied to correct the possible non-normality. Our primary and secondary outcomes are continuous and measured over three time points. We will use a longitudinal data analysis approach to asses within-group changes (change over time within each arm) and between-group changes (difference between two groups at each time point) while these assessments are adjusted for potential confounders (such as age, gender, ethnicity and employment status) and the correlation among the repeated measures. We will use a generalized estimation equation method with AR(1) (autoregressive) covariance structure or the random effect method to adjust for the correlation among the repeated measures of the participants. The longitudinal analysis also allows us to compare the change from the baseline at 12 and 24 weeks between intervention and control groups (difference in differences). The analysis will follow the intention-to-treat principle; all participants who are randomized will be included in the analyses and analyzed in the arms to which they were assigned. We will first analyze the data using observed data (complete case), then we impute the missing observations and obtain multiply imputed estimates. There are no prespecified subgroup analyses.

#### Implementation

We will use descriptive statistics to characterize the reach (recruitment sources and totals) and dose (hours of participation and hours of meditation practice per week). Interview and focus group data will be analyzed using a qualitative content analysis approach to develop and present a description of the phenomenon of interest. Interviews and focus groups will be transcribed verbatim and coded by two investigators to ensure consensus on emerging codes and categories. Emergent descriptive categories will be populated with data, and commonalities and discrepancies in participant responses noted. The coding framework will be refined based on additional emergent categories, and then used to further analyze and interpret the transcripts. This iterative process will continue as categories and constituent elements are developed, compared and contrasted between participant groups until a coherent description of participants’ perspectives is developed. Differing perspectives between groups will be represented in the results.

### Trial steering committee

A Trial Steering Committee will be struck with the following terms of reference: 1) to monitor and supervise progress of the RCT including risks and adverse events; 2) to review, at regular intervals, relevant information from other sources; and 3) to advise on dissemination, publicity and presentation of the results. The committee will also maintain the dated and versioned protocol to capture any amendments approved by the relevant research ethics boards and will update the trial registry accordingly. These amendments will be reported at the time of publication of final results. The committee chairperson will include one of the principal investigators. Additional membership will include a lay member of the public, a clinical trial expert who is not a co-investigator, the clinic medical director, and a knowledge user from the CMHA. Given the low-risk nature of the intervention and control we will not constitute a formal Data Safety and Monitoring Board, as approved by the research ethics boards.

### Dissemination plan

Study findings will be shared with participants and supporting staff at the clinic through letters of appreciation and group debriefing, respectively. Findings will also be presented at relevant conferences and published in relevant scientific journals. Integrated knowledge users and the collaborators CMHA and AOL are key to ensuring the success of a dissemination plan. CMHA is the most established and extensive community mental health organization in Canada and it has been active in advocating for improved mental health care. This study will help: 1) CMHA branches across Canada to integrate SSM into their existing programs and services; and 2) CMHA national and provincial leaders to advocate for greater access to meditation as a health intervention for chronic pain and depression. AOL is one of the world’s largest nongovernmental organizations with an active presence in over 150 countries. The organization works in a special consultative status with the United Nations Economic and Social Council and it leads a variety of humanitarian efforts relating directly to health and to social and environmental determinants of health. In Canada, AOL has over 200 active teachers from all provinces in 35 different chapters. They can help to disseminate evidence at the grassroots level; they can also be enlisted to provide community-based health-related meditation programming directed towards people with chronic pain. With over 5000 AOL teachers in over 150 countries, findings from this study would also have global reach.

## Discussion

To our knowledge, MEDOTATE will be one of the first RCTs examining meditation as an intervention for people living with chronic pain and significant depressive symptoms. It is the only such study to date to be designed and statistically powered for depression as a primary outcome. This is important because of the high prevalence of comorbid chronic pain and depression and because of the impacts of this comorbidity on individual and population health outcomes. It is even more important, however, because people living with depression and other mental illness comorbidities tend to be excluded from clinical trials examining physical health conditions, including trials for chronic pain [49, 50].

A structured diagnostic assessment is the preferred method for establishing a formal diagnosis of a depressive disorder. However, we have decided to use the PHQ-9 to establish the presence of clinically significant depressive symptoms. The PHQ-9 is a widely used clinical and research tool that has been well validated for this population. Thus, it is an appropriate screening and outcome measure for an effectiveness trial. Given our dual aims of establishing effectiveness and considering real-world implementation, it is appropriate to ground our analysis in a validated tool that will be much more likely to be used in community-based clinical settings where access to psychiatric services is limited [52].

Our choice to include patients with PHQ-9 ≥10 rather than patients with any depressive symptoms also represents a compromise between internal and external validity. Including patients with a higher depressive burden increases our knowledge about this understudied population and also increases the likelihood of measuring a statistically significant effect. It does, however, decrease the generalizability of our findings by not addressing the applicability of meditation for people with chronic pain and subthreshold depressive symptoms.

In terms of delivery model, we elected a co-location approach where community-based providers deliver the intervention at the physical premises (the clinic) where patients are recruited. There are both evidentiary and pragmatic reasons for this choice. Co-location has been shown to demonstrate effectiveness specifically for behavioral interventions [53]. Likewise, it is a more relevant model than collaborative care given that the interventionists for our study are not health professionals but instead are meditation teachers [54]. Pragmatically, we chose co-location so as to maximize recruitment and retention. Our belief is that providing a novel intervention in a familiar setting will make the intervention itself more accessible and familiar to patients. In addition, providing it in the bounds of a formal health care setting may help to improve perceptions of trustworthiness in the teachers and intervention.

This pragmatic rationale for co-location is important given the novelty of the intervention but also the novelty of doing research in this setting. The majority of chronic pain research has been conducted in tertiary academic settings that may not reflect community-based settings where the majority of health care is delivered. In many ways, a community-based chronic pain clinic may be an ideal setting in which to conduct clinical research into chronic pain. Patients in this setting are likely more representative of the larger population with chronic pain than the more complex patients who typically present in tertiary care settings. At the same time, there is a higher concentration of potentially eligible patients in specialized community settings than could be found in primary care. This evaluation will provide important insights of doing research in this novel setting. As an alternate delivery model, we considered recruiting patients from the clinic and referring them to an intervention site located in the community (e.g., a community center). However, we felt that this could significantly impair recruitment and participation in the program.

In terms of providers, the intervention will be provided by expert lay (not health care professional) meditation teachers. This is in contrast to current trends of increasing professionalization of meditation provision as a health care intervention. Our intention in doing so is to acknowledge that there are limitations in human health resources but there are also lay providers who are expert meditation teachers capable of and willing to provide meditation instruction to promote health and well-being. We hope this choice can facilitate scalability.

Finally, we must consider the intervention itself. For the purposes of a clinical trial, meditation programs should be standardized and protocolized to reduce variation between groups which can mediate effects. However, as with other behavioral interventions, it is important to acknowledge the socialized nature of the meditation instruction and practice. While it is relatively easy to reduce variation in RCTs assessing medications, it is more challenging to reduce variations in human interactions which depend more on time, space and context. For example, the life experiences of participants will be acknowledged and attended to during the meditation intervention. Different cohorts will have different sets of experiences that will determine the specific dynamics of how the intervention is delivered. Variation between groups cannot be completely eliminated. However, standardization and protocolization of intervention delivery can minimize this variation. These effects can be further minimized by randomization and an active control that is also a behavioral, group-based intervention.

## Supplementary information

**Additional file 1.** Completed World Health Organization Trial Registration Data Set.

**Additional file 2.** RCT Participant Informed Consent Form.

## Data Availability

Data sharing is not applicable to this article as no datasets were generated or analyzed at this point in time.

## Abbreviations

RCT: Randomized controlled trial
SSM: Sahaj Samadhi Meditation
HEP: Health Enhancement Program
PHQ-9: Patient Health Questionnaire
BPI: Brief Pain Inventory
LTOT: Long-term Opioid therapy
LTRI: Lunenfeld-Tanenbaum Research Institute
ES: Effect Size
AOL: Art of Living Foundation
CMHA: Canadian Mental Health Association
MINI: Mini International Neuropsychiatric Inventory
SD: Standard deviation
