# Supplementary material for: Sahaj Samadhi Meditation versus a Health Enhancement Program for depression in chronic pain: protocol for a randomized controlled trial and implementation evaluation
Source: Trials. 2020 Apr 7;21:319. doi: 10.1186/s13063-020-04243-z (PMC7140371; doi:10.1186/s13063-020-04243-z)
Supplement: Supplementary file 1 — Additional file 1. Completed World Health Organization Trial Registration Data Set. [file 13063_2020_4243_MOESM1_ESM.docx]

**Additional file 1: *Completed World Health Organization Trial Registration Data Set***

| **Data category** | **Information** |
| --- | --- |
| Primary registry and trial identifying number | ClinicalTrials.gov NCT04039568 |
| Date of registration in primary registry | 31 July, 2019 |
| Secondary identifying numbers | Mount Sinai Hospital Research Ethics Board: MSH 19-0163-A, University of Toronto Office of Research Ethics: 00038321, Canadian Institutes for Health Research Opioid Crisis Evaluation Grant: EO1-162072 |
| Source(s) of monetary or material support | Canadian Institutes for Health Research |
| Primary sponsor | Mount Sinai Hospital (Lunenfeld-Tanenbaum Research Institute – Bridgepoint Campus) |
| Secondary sponsor(s) | University of Toronto |
| Contact for public queries | *AS*, MD CCFP, abhimanyu.sud@utoronto.ca |
| Contact for scientific queries | Abhimanyu Sud, MD CCFP Department of Family and Community Medicine, Faculty of Medicine, University of Toronto; Institute of Health Policy, Management and Evaluation, Dalla Lana School of Public Health, University of Toronto |
| Public title | Meditation Versus Education for Improving Depression in Chronic Pain, a Randomized Controlled Trial |
| Scientific title | MEditation for Depression and Opioid use in chronic pain: an rcT And implemenTation Evaluation (MEDOTATE) |
| Countries of recruitment | Canada |
| Health condition(s) or problem(s) studied | Depression in chronic pain |
| Intervention(s) | Active control:*Health Enhancement Program* Intervention: *Sahaj Samadhi Meditation* |
| Key inclusion and exclusion criteria | Ages eligible for study: *>45 years* Sexes eligible for study: *both* Accepts healthy volunteers: *no*  Key inclusion criteria: *chronic pain (pain ≥3 months duration in any body region, by self-report); >45 years of age; on long-term opioid therapy (any opioid, at any dose for ≥3 months, by self-report); major depressive symptomology (PHQ-9 score ≥10); willing and able to attend all 4 training sessions of SSM/HEP, as well as 75% of follow-up sessions; understanding of English language; able to sit for 20–25 minutes without significant discomfort.*  Key exclusion criteria: *other major psychiatric condition including substance use disorder, psychosis, cognitive impairment, and imminent risk of suicide; that would practicing a form of mind-body intervention.* |
| Study type | Hybrid type 1 (effectiveness and implementation study) Allocation: *randomized* Intervention model: *parallel assignment* Masking: *single-blind (outcomes assessor)* Primary purpose: *intervention* |
| Date of first enrolment | October 2019 |
| Target sample size | 160 |
| Recruitment status | Recruiting |
| Primary outcome(s) | Change in depressive symptomology measured on the PHQ-9  Time frame: baseline, 12 weeks, 24 weeks |
| Key secondary outcomes | Change in pain severity measured on the Brief Pain Inventory (BPI), change in opioid dose, change in health-related quality of life measured on the Short Form 36  Time frame: baseline, 12 weeks, 24 weeks |
