# Supplementary material for: Sahaj Samadhi Meditation versus a Health Enhancement Program for depression in chronic pain: protocol for a randomized controlled trial and implementation evaluation
Source: Trials. 2020 Apr 7;21:319. doi: 10.1186/s13063-020-04243-z (PMC7140371; doi:10.1186/s13063-020-04243-z)
Supplement: Supplementary file 2 — Additional file 2. RCT Participant Informed Consent Form. [file 13063_2020_4243_MOESM2_ESM.docx]

**Additional file 2: *RCT Participant Informed Consent Form***

**Informed Consent – RCT Participants**

**Title** Alternative Treatments for Depression in Chronic Pain Study

**Investigator** Dr. Ross Upshur, MA MD MSc
Tel: 416-461-8252

**Co-Principal** Dr. Michelle Nelson, PhD

**Investigators** Dr. Abhimanyu Sud, MD CCFP

**Co-Investigators** Dr. Joel Katz, PhD CPsych
Dr. Rahim Moineddin, PhD
Dr. Benoit Mulsant, MD MS FRCPC
Dr. Akshya Vasudev, MBBS MRCPsych

**Study Staff** Mr. Darren K. Cheng, MSc

Ms. Alana Armas, MSc

**Funding** Canadian Institute of Health Research, Project Operating Grant: Evaluation of Interventions to Address the Opioid Crisis: non-pharmacological interventions for pain

**INTRODUCTION**

You are being asked to take part in a research study. Please read this explanation about the study and its risks and benefits before you decide if you would like to take part. You should take as much time as you need to make your decision. You should ask the study doctor or study staff to explain anything that you do not understand and make sure that all of your questions have been answered before signing this consent form. Before you make your decision, feel free to talk about this study with anyone you wish. Participation in this study is voluntary and your decision whether or not to participate will have no effect on your care at the Rivlin Medical Group Chronic Pain Clinic.

##### BACKGROUND AND PURPOSE

You are being considered for this study because you are over 45, have chronic pain treated with opioid medication, and may be feeling depressed. Depression is very common in people who have chronic pain. It is even more common in people who are prescribed opioids to help with their pain. Because people with chronic pain often already take a lot of medications, and since many medications can interact with opioids, we need to develop non-medication treatments. Also, we need treatments that can reach a lot of people because chronic pain and depression are so common and because there can be very long waits to see chronic pain specialists and psychiatrists. Past research has found that some non-medication treatments have good effects for depression and chronic pain. This research team showed that a low-cost program, taught by highly-trained but volunteer teachers, can reduce depression in older people. The program also helped reduce depression in people who had not been helped by medications. Most of the patients found the program easy to learn, and they tended to stick with the lessons. The program also improved the overall quality of life of the patients with depression. Both of these treatments will be offered in groups which could translate to cost-savings for the health care system.

We will build on this research experience to see if two easy-to-learn programs can help to improve depression symptoms in people with chronic pain and who take opioids. We will also look to see how these programs effect pain, function, medication use and quality of life. This research will help several large mental health and family medicine organizations understand how non-medication based treatments might help their clients. Results from this study will also be used to help support the development of health policy.

In this study we wish to explore two possible treatment options available for depression symptoms and chronic pain. Both of these treatments will be offered in groups which could translate to cost-savings for the health care system. The two treatment options are:

1. The Health Enhancement Program (HEP). Designed to give participants information and guidance on how to lead a healthy life-style.

2. Sahaj Samadhi Meditation (SSM). A unique and easy-to-learn meditation technique that reduces stress and provides deep relaxation.

The purpose of this study is to determine if HEP or SSM when combined with your existing treatments help relieve symptoms of depression as well as improve related symptoms like chronic pain. While both conditions may help, we do not know which condition may be better.

If you are eligible and willing to participate in the study, a computer program will be used to randomly assign you to either HEP or SSM, with an equal chance (50%) of assigning you to either program after the Screening Visit. You will be contacted by research staff to inform you of which program you will be attending and schedule your visits.

You are being invited to participate in this research study which will investigate two non-medication based treatment interventions for management of depression symptoms in people living with chronic pain.

STUDY DESIGN

All study assessments and visits will take place at the Rivlin Chronic Pain Clinic and scheduled based on your preference and availability. It is expected that you will be in the study for 12 weeks. During this time, you will attend a baseline assessment visit, 15 sessions of either HEP or SSM, followed by one focus group and a follow-up assessment visit, and a second follow-up assessment visit 6 months after you started the study. The baseline assessment will include questions about your general health and background to help describe you as a participant, and all assessment visits will include questions about your mood, pain, and quality of life. A focus group may also be completed at the completion of the 12-week program.

Whether you participate in the HEP or SSM program will be decided randomly (by chance) like flipping a coin or rolling dice. The number of people in each group is 80 and you will have a 50/50 chance of being assigned to either group.

You will continue with your usual treatments including medications and talk therapies, if you use such therapies during the study. This study is a single blind randomized controlled trial. This means that the study doctor and most study staff will not know if you are attending SSM or HEP until the study is finished. Only you and the instructor of your SSM or HEP group will know which program you are attending.

##### STUDY VISITS AND PROCEDURES

**Screening Visit:** If you are interested, able, and agree to participate in this study then you will be invited to attend a screening visit with our research team where you will will have the opportunity to ask any questions you may have. This visit also involves completing a one-to-one interview and completing some standardised questionnaires. It should take around half an hour to complete this screening assessment.

In order to determine if you are eligible for this study, the following questionnaires will be completed:

*Mini International Neuropsychiatric Interview (MINI).* A brief structured interview that assesses the 17 most common disorders in mental health. The MINI will be used to evaluate you for disorders that may exclude you from participating in the study and will take about 15 minutes to complete.

*Patient Health Questionnaire (PHQ-9).* A short questionnaire to screen and measure severity of depression symptoms. The PHQ-9 will be used to evaluate you for significant depression symptoms and takes about 5 minutes to complete

At the end of the screening visit we will be able to let you know if you qualify to participate in this study.

**Study Assessment Visits:** You will be invited to attend 3 total study assessment visits (The Baseline Assessment, and two Follow-up Assessments) at the Rivlin Medical Group in Mississauga, Ontario. These study visits will be used to help us determine if your mood and chronic pain have gotten better, worse, or stayed the same throughout the study to see how well HEP and SSM work.

At each assessment you will be asked a variety of questions. Some questions will be verbally asked by a member of the research team, and others will be given to you as a written questionnaire. You will also be instructed to bring your medication log, which you will be provided with at the beginning of the study and a list of your medications.

In this study, the **Baseline Assessment Visit** is a separate visit from your Screening Visit, and will take place before you begin your treatment program and will last about 45 minutes. At this visit, the following information will be collected:

You will be asked questions about yourself such as your medication history, age, gender, ethnicity, caffeine, drinking and smoking habits.

*Medication Log.* You will be asked about the medications you are currently taking, including doses and frequencies and if any were taken as needed.

*PHQ-9* as described above.

*Brief Pain Inventory (BPI).* A short questionnaire that assesses the severity of pain and its impact on functioning and takes about 5 minutes to complete.

*Quality of Life measurement (SF-36).* A questionnaire that measures functional health, quality of life and well-being that takes about 10 minutes to complete.

**Treatment Interventions – HEP or SSM:** Both HEP and SSM require the same time involvement. Both programs will be taught in groups of 10-15 participants and require a training week where you will attend 2-hour sessions, four days in-a-row. The training week will then be followed by weekly 75 minute reinforcement sessions for the next 11 weeks. You will be asked to complete daily homework after the first week, which is expected to take 40 minutes per day to complete, and you will be asked to keep a log indicating how frequently you completed the homework. We will make all efforts to ensure that the timing and location of these sessions are convenient to most of the study participants. However, it is possible that these do not match your own availability or convenience. If that is so, please do inform the Research Coordinator and alternative dates or program start times can be arranged.

The Health Enhancement Program (HEP). The HEP provides information to lead a healthy life. This includes education on healthy diet, exercise, and relaxation through music. This program offers the support of a group and facilitator to assist with talking through ways to implement a positive healthy life.

Sahaj Samadhi Meditation (SSM). SSM involves relaxed attention to a precise sound (mantra), to allow stress reduction and deep relaxation. This technique is easy to learn. Similar to HEP, SSM offers the support of a group and facilitator.

Instructors from the Art of Living Foundation will be providing the SSM programs under the supervision of Ms. Ronnie Newman.

Please know that most study staff and the study doctor will **not** know if you are in the SSM group or the HEP group. *You are asked to not let the study staff or study doctor know which group you are attending during assessment visits.*

**Focus Group:** You may be invited to attend a one-hour focus group after your 15^th^ session to discuss your experience in the study. This focus group will be led by a research staff member who has experience leading focus groups. It will provide those participating with an opportunity to discuss what they did and did not like about the program and suggest possible improvements. The focus groups will be audio-recorded and transcribed. Personal and confidential information will be removed from the transcripts.

**Follow-up Assessment Visits 1 and 2:** Follow-Up Assessment Visit 1 will take place after your final (15th) HEP or SSM session, 12 to 13 weeks after your first assessment. Follow-up Assessment Visit 2 will take place 6 months (24 weeks) after your first assessment. Both *Follow-up Assessment Visits* will last about 30 minutes.

At both of these assessment visits, the *Medication Log, PHQ-9, BPI, and SF-36* will be completed as described above for the Baseline Assessment Visit.

**CALENDAR OF VISITS**

**Boxes marked with an X show what will happen at each visit:**

| Data Collected or Treatment | **Screening** | **Baseline Assessment** | **Initial Training**  **(Week 1)** | **Reinforcement Sessions (Week 2 to 12)** | **Focus Group**  **(Week 12)** | **Follow-up Assessment 1**  **(Week 12/13)** | **Follow-up Assessment 2**  **(week 24/25)** |
| --- | --- | --- | --- | --- | --- | --- | --- |
| *Time* | *30 mins* | *45 mins* | *2 hours x 4* | *75 mins x 11* | *1 hour* | *30 mins* | *30 mins* |
| Informed Consent | X |  |  |  |  |  |  |
| Appropriateness for study | X |  |  |  |  |  |  |
| Mini International Neuropsychiatric Interview (MINI) | X |  |  |  |  |  |  |
| Patient Health Questionnaire (PHQ-9) | X | X |  |  |  | X | X |
| Demographic information |  | X |  |  |  |  |  |
| Brief Pain Inventory (BPI) |  | X |  |  |  | X | X |
| Quality of Life measurement (SF-36) |  | X |  |  |  | X | X |
| Medication Information |  | X |  |  |  | X | X |
| HEP or SSM |  |  | X | X |  |  |  |
| Focus Group |  |  |  |  | X |  |  |

##### REMINDER

It is important to remember the following things during this study:

- These programs are not replacements for your current treatment – you should continue your regular treatment as directed by your physician.
- Ask the study team about anything that worries you.
- Tell study staff anything about your health that has changed.
- Tell your study team if you change your mind about being in this study.
- Tell study staff if you cannot attend a scheduled session.

### RISKS RELATED TO BEING IN THE STUDY

While rare, the most commonly reported adverse effects of participating in SSM include mild anxiety caused by the relaxation itself. Other adverse effects that may be experienced include boredom and feeling mildly detached from one’s surroundings. There are no known or anticipated risks or discomforts associated in participating in the HEP. This study carries no risks related to pregnancy.

If you experience any side effects other than those listed above, you will be advised to consult with your primary care physician and asked to inform study staff. Study staff will consult with the Investigators to determine if after experiencing such side effects, you should continue with the study. Your participation in the study will stop if it is considered medically unsafe to continue and you will be referred for further clinical management of the side effect.

The questionnaires and scales used in the study carry little risk. You may feel uncomfortable, sad, embarrassed or anxious as some of the questions you will be invited to answer are personal. You may refuse to answer questions or stop the interview at any time if there is any discomfort.

Any indication of a change in your overall mental health/risk of suicide will be reported immediately to the Principal Investigator for further clinical assessment and management. If you are considered an imminent risk you will be referred to the staff at Rivlin Medical Group.

### BENEFITS TO BEING IN THE STUDY

Your participation will help to improve the understanding of treatments that might be helpful in reducing depression symptoms and chronic pain in the future. You may experience reduction in depressive and pain symptoms. There is also the possibility that you will receive no personal benefit from this study.

### INCIDENTAL FINDINGS

Questionnaires for the study are not for the purpose of diagnosis, and results will not be reviewed by a doctor, or recorded in your medical history. However, a potential risk of participating in research is that investigators may discover an abnormality of uncertain significance that may impact on your health. If warranted, a physician from the clinic will meet with you to discuss the findings, and provide you with a letter to take to your family physician describing the circumstances of the findings, and a recommended course of action. Your family physician may, in turn, recommend further diagnostic tests, which may then have an impact on your health and insurance.

The study Principal Investigator may choose to withdraw you from the study at anytime for reasons including but not limited to your safety and/or health.

### VOLUNTARY PARTICIPATION

Your participation in this study is voluntary. You may decide not to be in this study, or to be in the study now and then change your mind later. You may leave the study at any time without affecting your care. You may refuse to answer any question you do not want to answer, or not answer an interview question by saying “pass”.

If you withdraw from the study any information that was collected prior to when you leave the study will still be used for the study unless you request otherwise. No new information will be collected from you. The study Principal Investigator may choose to withdraw you from the study at anytime for reasons including but not limited to your safety and/or health. We will give you new information that is learned during the study that might affect your decision to stay in the study.

##### ALTERNATIVES TO BEING IN THE STUDY

You do not have to join this study to receive treatment for your condition. The programs in this study are being tested as alternatives to medication and therapies, but does not require you to stop the use of other treatments.

**CONFIDENTIALITY**

If you agree to join this study, the study doctor and his/her study team will collect only the information they need for the study. The information that is collected for the study will be kept in a locked and secure area by the study doctor for 7 years or on a secure online database. Only the study team or representatives of the Mount Sinai Hospital Research Ethics Board may look at the study records and at your personal health information to check that the information collected for the study is correct and to make sure the study followed proper laws and guidelines.

To further protect your confidentiality, your name will be replaced with a participant ID number on all documents. The master list linking your identity and participant ID number will be stored electronically for 7 years on a secure database at the Bridgepoint Collaboratory. If the results of the study are published, your name will not be used and no information that discloses your identity will be released or published. Your results will remain de-identified and will be combined with those of other participants. No information that could reveal your identity will be released to anyone.

The rare exception to guaranteeing confidentiality is in cases where you indicate you may harm yourself or someone else. Then we are required to disclose information by law.

As HEP and SSM are group therapies conducted in groups of 10 to 15 participants, there may be a risk to your confidentiality associated with participating in a group. Neither study staff nor program instructors will disclose any personal information to other participants. However, information shared by you within the group cannot be guaranteed to be confidential.

If you are participating in another study at this time, please inform the study coordinator right away to determine if it is appropriate for you to participate in this study.

Personal information we will be collecting about you for this study will include:

• Your first and last name

• Your age

• Your gender

• Your telephone number, e-mail and/or mailing address for contact purposes

##### CLINICAL TRIAL REGISTRATION

A description of this clinical trial will be available on <http://www.ClinicalTrials.gov>, as required by U.S. Law. This Web site will not include information that can identify you. At most, the Web site will include a summary of the results. You can search this Web site at any time.

##### IN CASE YOU ARE HARMED IN THE STUDY

If you become ill, injured or harmed as a result of taking part in this study, you will receive care. The reasonable costs of such care will be covered for any injury, illness or harm that is directly a result of being in this study. In no way does signing this consent form waive your legal rights nor does it relieve the investigators, sponsors or involved institutions from their legal and professional responsibilities. You do not give up any of your legal rights by signing this consent form.

**EXPENSES ASSOCIATED WITH PARTICIPATING IN THE STUDY**

You will be offered the opportunity to participate in SSM or HEP at no cost to you, but you will not be otherwise compensated based on your attendance of SSM or HEP. You will be responsible for arranging transportation to and from your 3 assessment visits and 15 SSM or HEP sessions including any costs incurred for transportation such as bus fare, gas, or parking.

##### CONFLICT OF INTEREST

Costs of doing this study are being funded by the Canadian Institute of Health Research. These people have an interest in completing this study. Their interests should not influence your decision to participate in this study. You should not feel pressured to join this study.

**QUESTIONS ABOUT THE STUDY**
If you have any questions, concerns or would like to speak to the study team for any reason, please call the study Research Coordinator **Darren Cheng** at 416 461 8252 ext. 2884 or send an email to [darren.cheng@sinaihealthsystem.ca](mailto:darren.cheng@sinaihealthsystem.ca)

If you have any questions about your rights as a research participant or have concerns about this study, call Ronald Heslegrave, Ph. D., Chair of the Mount Sinai Hospital Research Ethics Board (REB) or the Research Ethics office number at 416-586-4875. The REB is a group of people who oversee the ethical conduct of research studies.These people are not part of the study team. Everything that you discuss will be kept confidential.

### CONTACT FOR FUTURE STUDIES

Please check the appropriate box below and initial:

I agree to be contacted for future research studies

I DO NOT agree to be contacted for future research studies

### CONSENT

This study has been explained to me and any questions I had have been answered.

I know that I may leave the study at any time. I agree to take part in this study and to the use of my personal health information as described above.

Print Name of Participant Signature Date (dd-mm-yyyy)

(You will be given a signed copy of this consent form)

My signature means that I have explained the study to the participant named above. I have answered all questions.

Print Name of Person Signature Date (dd-mm-yyyy)
Obtaining Consent
